# CT-like images based on T1 spoiled gradient-echo and ultra-short echo time MRI sequences for the assessment of vertebral fractures and degenerative bone changes of the spine

**DOI:** 10.1007/s00330-020-07597-9

**Published:** 2021-01-14

**Authors:** Benedikt J. Schwaiger, Charlotte Schneider, Sophia Kronthaler, Florian T. Gassert, Christof Böhm, Daniela Pfeiffer, Thomas Baum, Jan S. Kirschke, Dimitrios C. Karampinos, Marcus R. Makowski, Klaus Woertler, Markus Wurm, Alexandra S. Gersing

**Affiliations:** 1 Department of Diagnostic and Interventional Radiology, Klinikum rechts der Isar, School of Medicine, Technical University of Munich, Munich, Germany; 2 Department of Diagnostic and Interventional Neuroradiology, Klinikum rechts der Isar, School of Medicine, Technical University of Munich, Munich, Germany; 3 Department of Trauma Surgery, Klinikum rechts der Isar, School of Medicine, Technical University of Munich, Munich, Germany

**Keywords:** Magnetic resonance imaging, Spine, Degenerative intervertebral discs, Spinal fractures

## Abstract

**Objectives:**

To evaluate the performance of 3D T1w spoiled gradient-echo (T1SGRE) and ultra-short echo time (UTE) MRI sequences for the detection and assessment of vertebral fractures and degenerative bone changes compared with conventional CT.

**Methods:**

Fractures (*n* = 44) and degenerative changes (*n* = 60 spinal segments) were evaluated in 30 patients (65 ± 14 years, 18 women) on CT and 3-T MRI, including CT-like images derived from T1SGRE and UTE. Two radiologists evaluated morphological features on both modalities: Genant and AO/Magerl classifications, anterior/posterior vertebral height, fracture age; disc height, neuroforaminal diameter, grades of spondylolisthesis, osteophytes, sclerosis, and facet joint degeneration. Diagnostic accuracy and agreement between MRI and CT and between radiologists were assessed using crosstabs, weighted κ, and intraclass correlation coefficients. Image quality was graded on a Likert scale.

**Results:**

For fracture detection, sensitivity, specificity, and accuracy were 0.95, 0.98, and 0.97 for T1SGRE and 0.91, 0.96, and 0.95 for UTE. Agreement between T1SGRE and CT was substantial to excellent (e.g., Genant: *κ*, 0.92 [95% confidence interval, 0.83–1.00]; AO/Magerl: *κ*, 0.90 [0.76–1.00]; osteophytes: *κ*, 0.91 [0.82–1.00]; sclerosis: *κ*, 0.68 [0.48–0.88]; spondylolisthesis: ICCs, 0.99 [0.99–1.00]). Agreement between UTE and CT was lower, ranging from moderate (e.g., sclerosis: *κ*, 0.43 [0.26–0.60]) to excellent (spondylolisthesis: ICC, 0.99 [0.99–1.00]). Inter-reader agreement was substantial to excellent (0.52–1.00), respectively, for all parameters. Median image quality of T1SGRE was rated significantly higher than that of UTE (*p* < 0.001).

**Conclusions:**

Morphologic assessment of bone pathologies of the spine using MRI was feasible and comparable to CT, with T1SGRE being more robust than UTE.

**Key Points:**

*• Vertebral fractures and degenerative bone changes can be assessed on CT-like MR images, with 3D T1w spoiled gradient-echo–based images showing a high diagnostic accuracy and agreement with CT.*

*• This could enable MRI to precisely assess bone morphology, and 3D T1SGRE MRI sequences may substitute additional spinal CT examinations in the future.*

*• Image quality and robustness of T1SGRE sequences are higher than those of UTE MRI for the assessment of bone structures.*

## Introduction

Pathologies of the spine are among the greatest contributors to morbidity and mortality worldwide. In particular, degenerative changes such as intervertebral disc degeneration or degenerative spondylolisthesis have a lifetime prevalence of 60–90% [1–4]. Osteoporosis, either as a separate entity or in conjunction with degenerative changes, is another highly relevant pathology with a continuously increasing prevalence [5, 6]. Ultimately, prevalent vertebral and hip fractures led to an increased risk of mortality 5 to 10 years after the fracture event, respectively [7, 8].

Both in patients with vertebral fractures and those with degenerative changes, CT and MR imaging are often performed [9]. In patients with fractures, this is done to differentiate acute from chronic fractures and to assess the surrounding soft tissues [10, 11]. In patients with degenerative changes, this is done since pathologies of the spine—analogously to most other musculoskeletal entities—comprise both, a soft-tissue and an osseous component. CT examinations of the spine are associated with radiation exposure, additional examination time, and costs [12, 13].

Obtaining all relevant information on soft tissue as well as osseous structures within the same examination would therefore be highly desirable. While for the general detection of bone pathologies, e.g. cellular infiltration and sclerosis in bone metastasis, a fast and large field-of-view 3D T1w may be the best MRI sequence [14], high-resolution sequences with bone-specific signal characteristics may be the technique of choice for the precise assessment of bone morphology. For this, several approaches based on MRI have been previously suggested: “Simulated” CT images based on a 3D T1w fast-field echo MR sequence showed a high agreement with CT for the assessment of glenoid bone loss [15]. Similarly, it has been suggested that images resembling radiographs based on bone surface models derived from 3D MRI data allow for anatomic measurements in the ankle [16]. Recently, “simulated” radiographs and CT-like images were generated based on a 3D T1w spoiled gradient-echo MR sequence, in order to evaluate bone destruction patterns and periosteal reactions. The latter study showed that the accurate evaluation of bone tumors was possible using these images [17]. In other recent studies, the use of ultra-short echo time (UTE) and zero echo time (ZTE) sequences for the depiction of cortical and trabecular bone has been suggested [18–20]. UTE and ZTE sequences were successfully applied to other regions of the musculoskeletal system [21], but their application remains challenging in the spine due to folding and motion artifacts.

The purpose of this study therefore was to evaluate the diagnostic performance of MR-derived CT-like images based on high-resolution 3D T1w spoiled gradient-echo (T1SGRE) and ultra-short echo time (UTE) sequences for the identification and morphological assessment of vertebral fractures and degenerative bone changes in the thoracolumbar spine compared with conventional CT as a standard of reference.

## Methods

### Patient selection

An institutional review board (IRB) approval was obtained prior to this study (Ethics Commission, School of Medicine, Technical University of Munich, Germany). Patients gave their written informed consent.

Between December 2018 and October 2019, consecutive patients admitted to our emergency department were screened for study participation. Inclusion criteria were (i) admission for suspected acute thoracolumbar vertebral fracture, (ii) a CT acquired as part of the routine clinical diagnostic work-up, and (iii) feasibility of MR imaging within 3 days. Of 79 potentially eligible patients that were available for initial assessment regarding their participation in the study, 49 had to be excluded for the following reasons: 9 had contraindications for MR imaging (e.g., a pacemaker), 8 had undergone surgery immediately after the CT examination and therefore could not be examined with MR imaging, and 32 patients chose not to participate.

From our clinical information system, demographic data and clinical history including recent falls and symptoms were obtained.

### CT imaging

CT was performed on one of two CT scanners (Somatom Definition AS+, Siemens Healthineers, and IQon Spectral CT, Philips) with the following parameters, according to routine clinical protocols: collimation, 0.6 mm; pixel spacing, 0.4/0.3 mm; pitch factor, 0.8/0.9; tube voltage (peak), 120 kV; modulated tube current, 102–132 mA. Images were reformatted in 3-mm slice thickness using a bone-specific convolution kernel (I70H/YB).

### MR imaging and post-processing

Within 3 days, MR imaging was performed on one 3-T scanner (Ingenia Elition, Philips) using 16-channel anterior and posterior coils. A 3D T1-weighted partial spoiled gradient echo (T1SGRE) and a 3D UTE sequence were added to the routine spine protocol (sequence parameters for T1SGRE and UTE, see Table 1). For this, a 3D UTE stack-of-stars sequence was employed with a non-selective RF pulse and Cartesian phase encoding in the third dimension [22] in order to assess the signal of tissues with short T2*. Of note, due to the hard RF pulse of the UTE sequence, a larger FOV in the slice direction had to be used to avoid folding artifacts. As soon as possible after the excitation, the FID was acquired in-plane along one center-out radial “spoke”. The non-Cartesian trajectories were estimated using the gradient impulse response function of the system. For the reconstruction, an image reconstruction toolbox (ReconFrame, Gyrotools) was used to grid the data in two dimensions with the corresponding k-space trajectories and to Fourier transform in 3D.

**Table 1:** Sequence parameters for 3D T1-weighted spoiled gradient echo (T1SGRE) and ultrashort echo (UTE) sequences

	T1SGRE	UTE
Echo time (ms)	2.3	0.14
Repetition time (ms)	7.8	6.3
Flip angle	8°	5°
Field of view (craniocaudal, anterior-posterior, left-right; mm)	250 × 160 × 70	250 × 259 × 279
Voxel size (acquisition; mm)	0.45 × .045 × 1.5	0.45 × 0.45 × 3
Voxel size (reconstruction; mm)	0.28 × 0.28 × 0.75	0.28 × 0.28 × 0.75
Parallel imaging	None	None
Comment	Partial Fourier imaging in frequency encoding direction (60%)	3D stack-of-stars sequence with non-selective RF pulse and Cartesian phase encoding in the third dimension.
Acquisition duration (average ± standard deviation; min)	5.12 ± 0.17	6.3 ± 0.23

**Fig. 1:**
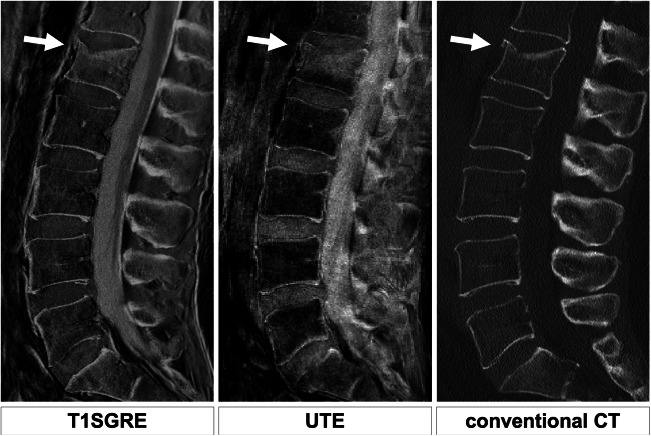
Comparison of T1SGRE-derived CT-like images (left), UTE images (middle), and conventional CT images (right) of the same patient. While the acute compression fracture of L1 (Genant °I, AO/Magerl A1) can be identified and classified in all modalities; the T1SGRE sequence shows a more homogeneous signal and tissue contrast optimal for the assessment of osseous structures. Of note, due to the inverted-grayscale T1w contrast, ligaments are also shown as hyperintense which must not be misinterpreted as calcifications

**Fig. 2:**
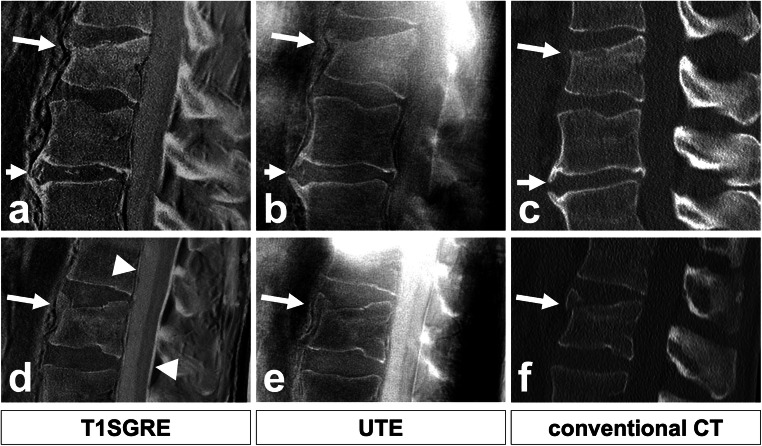
Comparison of T1SGRE-derived CT-like images (**a**, **d**), UTE images (**b**, **e**), and conventional CT images (**c**, **f**). In one patient (**a**–**c**), a wedge-compression fracture of L1 with signs of an acute pathology such as a compaction zone can be depicted (upper arrows), as well as ventral and a small dorsal osteophytes on level L2/3 (lower arrows). In another patient (**d**–**f**), another wedge-compression fracture of L2 with a triangular teardrop-like fragment can be identified (arrows). Also note the thin hyperintense line running longitudinally along the posterior walls of vertebral bodies representing the posterior longitudinal ligament as well as the thicker hyperintense line posterior to the dural sac representing the ligamenta flava (arrowheads; **d**), which are not depicted on CT (**f**), and must not be misinterpreted as ligament calcifications

For the evaluation of osseous structures, both the 3D T1w GRE and UTE sequences were reformatted in sagittal, coronal, and axial orientation with a slice thickness of 3 mm, grayscales were inverted, and windowing was set to resemble a CT bone window (Figs. 1 and 2).

According to our clinical standard for suspected vertebral fractures and degenerative changes, the protocol further comprised a sagittal short-tau inversion recovery (STIR) sequence, sagittal T1w and T2w spin-echo sequences, and a transversal T1w spin-echo sequence.

### Image analysis

MR and CT images were individually and independently read by two radiologists (B.J.S., a board-certified radiologist with 10 years of experience in MSK imaging and F.T.G., a radiology resident), blinded to all other information including clinical and results from other modalities, including other MRI sequences when evaluation the bone-specific T1SGRE and UTE sequences. The images were read in a randomized order. Image evaluation and quantitative measurements were performed on a PACS certified for clinical use (IDS7 21.2, Sectra). Between T1SGRE, UTE, and CT imaging readings, there was an interval of at least 8 weeks, respectively.

**Fig. 3:**
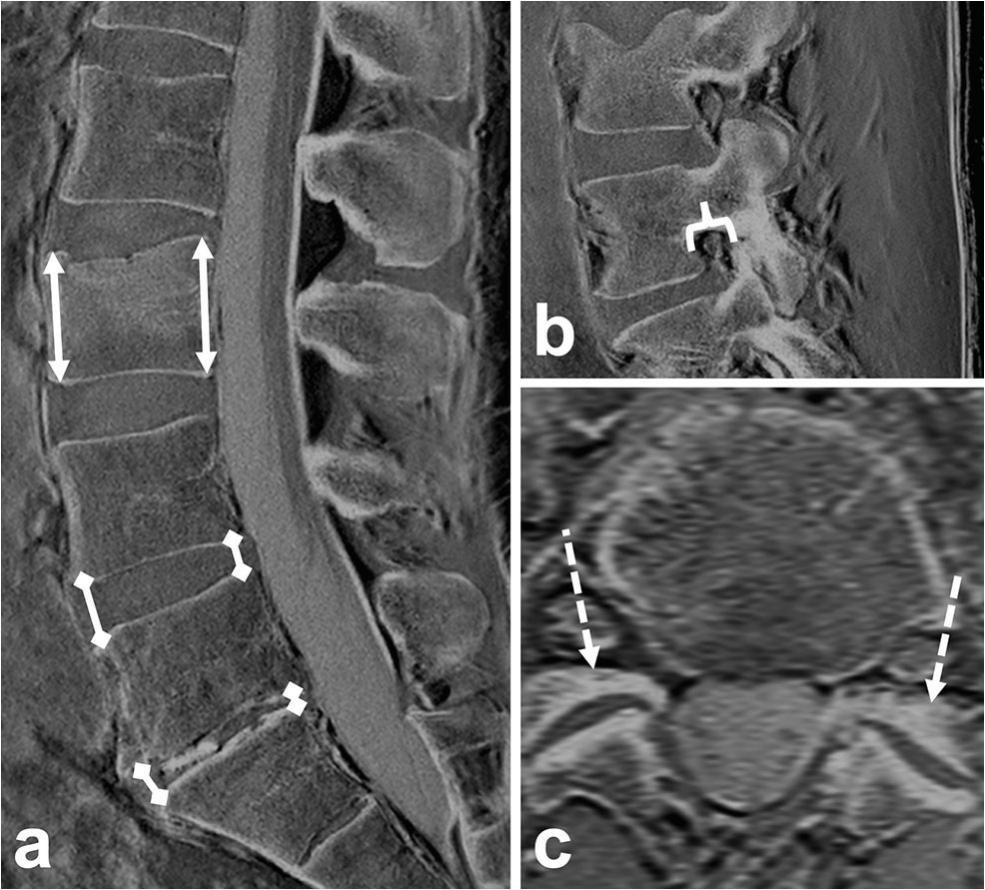
Exemplary measurements in a 63-year-old male patient with an acute wedge-compression fracture of L3: Anterior and posterior height of the vertebral body (**a**; arrows); anterior and posterior height of intervertebral discs (**a**; arrows with rhomboid tips); neuroforaminal AP diameter (**b**; bracket); extent of facet joint degeneration (here: joint space narrowing + sclerosis; **c**; dashed arrows)

First, the presence and location of vertebral fractures were assessed and noted. Then, the following morphological features were evaluated using a standard template (Table 2; Fig 3): height of the anterior and posterior vertebral edge in the mid-sagittal plane, height loss according to Genant et al [23], fracture classification according to AO/Magerl [24, 25], and differentiation of acute vs. chronic vertebral fractures according to Hedderich et al [26]. In patients with more than one fracture, each level was evaluated separately.

**Table 2:** Imaging parameters for the assessment of vertebral fractures and degenerative changes

Parameter	Description and references	Grading and frequency distribution (*n*, %) or mean ± standard deviation*
Fractures		
Genant classification	Semiquantitative visual grading of vertebral deformities according to Genant et al [23]	Grade 1 (20–25% reduction in height): 23 (52%)
		Grade 2 (25–40%): 12 (27%)
		Grade 3 (> 40%): 9 (21%)
Anterior height vertebral body	Measured in the median sagittal plane, from the anterosuperior to the anteroinferior corner of the vertebral body, excluding osteophytes, or dislocated fragments	19.4 ± 5.5 mm
Posterior height vertebral body	Measured in the median sagittal plane, from the posterosuperior to the posteroinferior corner of the vertebral body, excluding osteophytes, or dislocated fragments	24.8 ± 4.0 mm
AO/Magerl fracture classification	Classification of fractures in compression, distraction, and translation injuries according to Magerl et al [24] and Vaccaro et al [25]	A1 (wedge compression): 30 (68%)
		A2 (split): 1 (2%)
		A3 + 4 (incomplete and complete burst): 13 (30%)
		B (distraction): 0
		C (displacement or dislocation): 0
Fracture age	Classification of fractures in acute and chronic fractures according to Hedderich et al [26]	Definitely chronic: 14 (33%)
		Likely chronic: 1 (2%)
		Likely acute: 11 (25%)
		Definitely acute: (40%)
Degenerative changes		
Anterior disc height	Measured in the median sagittal plane, from the anteroinferior to the anterosuperior corner of the vertebral body, analogously to Frobin et al [27]	10.7 ± 5.6 mm
Posterior disc height	Measured in the median sagittal plane, from the posteroinferior to the posterosuperior corner of the vertebral body, analogously to Frobin et al [27]	5.5 ± 5.2 mm
Neuroforaminal AP diameter	Minimum AP diameter of the narrower side, measured in sagittal reformations according to Mamisch et al [28]	9.2 ± 2.0 mm
Spondylolisthesis	Maximum extent of listhesis, measured in the median sagittal plane	0.5 ± 2.1 mm
Osteophytes	Anterior, lateral, and posterior osteophytes, assessed analogously to Wilke et al [29]	None: 24 (41%)
		Mild: 25 (43 %)
		Moderate: 8 (14%)
		Severe: 1 (2%)
Sclerosis	Extent of diffuse sclerosis in adjacent vertebral bodies, analogously to Wilke et al [29]	None: 38 (66%)
		Partially in one vertebra: 9 (16%)
		Partially in both vertebrae or encompassing the whole cross-sectional area in one vertebra: 9 (16%)
		Encompassing the whole cross-sectional area in both vertebrae: 2 (3%)
Facet joint degeneration	Extent of facet joint degeneration (if asymmetric, the more severe side was noted), according to Weishaupt et al [30]	Normal facets: 17 (29%)
		Joint space narrowing: 25 (43%)
		Plus sclerosis or hypertrophy: 15 (26%)
		Severe degeneration with narrowing, sclerosis and osteophytes: 1 (2%)
		Plus synostosis: 0

*As shown by conventional CT as a standard of reference

Furthermore, in every patient, in the two non-fractured segments with the most prominent degenerative changes (as determined by the more experienced radiologist, B.J.S.), the following imaging findings were evaluated (Table 2; Fig 3): anterior and posterior distance between bony endplates (i.e., intervertebral disc height) [27], anteroposterior (AP) diameter of intervertebral foramina [28], spondylolisthesis (distance between vertebral body rims, mm), and extent of diffuse sclerosis adjacent to one or both vertebral endplates [29], of osteophyte formation [29], and of facet joint degeneration [30].

Images from all modalities were graded for overall diagnostic image quality on a five-point Likert scale (score of 1, inadequate; 2, poor; 3, moderate; 4, good; 5, excellent).

### Statistical analysis

In addition to descriptive statistics, the agreement of numerical, approximately normally distributed data was evaluated with intraclass correlation coefficients (ICC) and Bland-Altman plots were created for illustration purposes. The diagnostic performance of MRI for the detection of fractures was assessed using contingency tables. The agreement of ordinal scaled parameters was assessed using weighted Cohen’s *κ* [31]. To assess inter-reader reproducibility of the readings of MR-based CT-like images and CT images, the same tests were used. For all measures, 95% confidence intervals (CI) were calculated. B.J.S. (11 years of experience in biostatistics) analyzed all data with SPSS, version 25 (IBM).

## Results

### Patient characteristics, morphology, and image quality

In total, 30 patients (65 ± 14 years; 60% female) with a total of 44 vertebral fractures (according to CT as the standard of reference) were included in this analysis, and on average, each patient had 1.5 vertebral fractures (range, 1–4). Of the fractures, 25 were considered acute according to the presence of edema-like signal alterations on STIR as well as clinical symptoms. Fractures were most often found in L1 and L2 (each, *n* = 10), followed by Th12 (*n* = 9), L3 (*n* = 6), and the remaining thoracolumbar vertebral bodies. According to CT as the standard of reference, the majority of fractures were classified as wedge-compression fractures (AO A1; 68%) and incomplete and complete burst fractures (AO A3 + 4; 30%; Table 2). Degenerative changes were assessed in two non-fractured vertebral segments in each patient (*n* = 60) and ranged from “no degenerative changes present” to “severe degenerative changes present” (Table 2).

The median for rating diagnostic quality of T1SGRE-derived images was 5 (excellent), with 59% of cases rated as excellent, 24% as good, 10% as moderate, 7% as poor, and none as inadequate (Figs. 1 and 2). The diagnostic quality of UTE images was rated significantly lower (median, 3 (moderate); with 45% of cases rated as good, 31% as moderate, and 24% as poor; *p* < 0.001). The median for rating diagnostic quality of conventional CT images was 5 (excellent), with 82% of cases rated as excellent, and 17% as good.

### Diagnostic performance of MRI and agreement of MR and CT images

On the T1SGRE sequence, 42 (reader 1) and 41 (reader 2) of 44 fractures were detected, using CT as standard of reference, while 3 (reader 1) and 3 (reader 2) fractures were considered to be false-positive vertebral fractures (sensitivity, 0.95/0.93; specificity, 0.98/0.98; accuracy 0.97/0.97 for radiologist 1 and 2, respectively). On the UTE sequence, 40 (reader 1) and 38 vertebral fractures (reader 2) were correctly identified, and 5 (reader 1) and 6 vertebral fractures (reader 2) were false-positive vertebral fractures (sensitivity, 0.91/0.86; specificity, 0.96/0.96; accuracy 0.95/0.94 for radiologist 1 and 2, respectively).

**Fig. 4:**
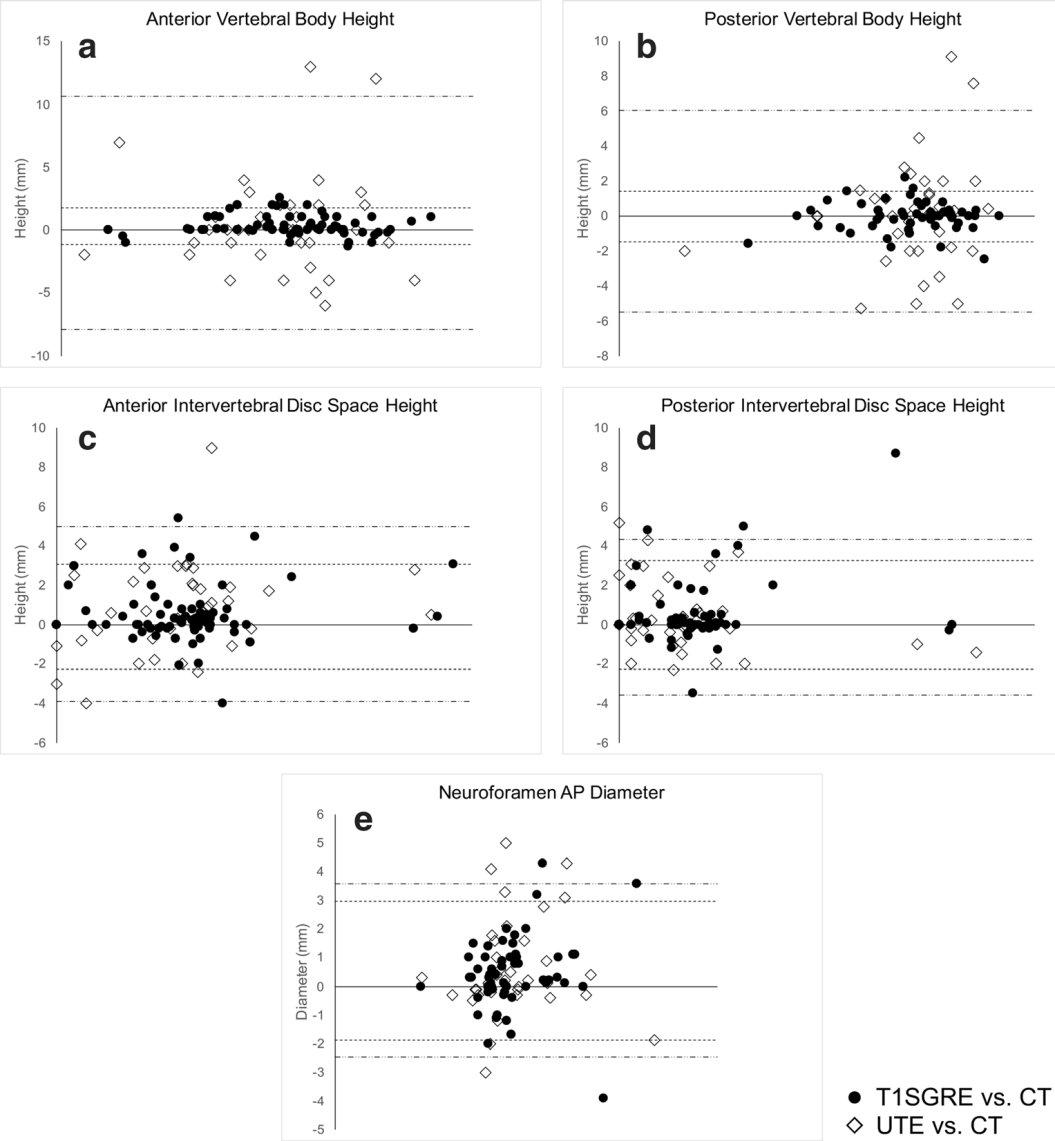
Bland-Altman plots for agreement between quantitative measurements on T1SGRE/ UTE and CT images, respectively. Measurements on T1SGRE vs. CT images are shown as black dots, and upper and lower limits of agreement are marked with fine dashed lines, respectively. Measurements on UTE vs. CT images are shown as white rhombi, and upper and lower limits of agreement are marked with alternately dashed and dotted lines, respectively

Between T1SGRE-derived CT-like images and CT, agreement for quantitative parameters such as anterior and posterior heights of the vertebral body was excellent (ICCs, 0.99 [0.99–1.00], respectively (both radiologists); Table 3, Fig. 4). The agreement for ordinal-scale parameters was excellent as well, ranging between *κ* 0.81 (0.71–0.92) for fracture age (radiologist 2) and *κ* 0.92 (0.83–1.00) for Genant classification (both radiologists; Table 3). For quantitative parameters describing degenerative changes, agreement was excellent, ranging between ICC 0.81 (0.62–0.90) for neuroforaminal AP diameter (radiologist 2) and 1.00 (1.00–1.00) for spondylolisthesis (both radiologists; Table 3, Fig. 4). The agreement for ordinal-scale parameters ranged between substantial (sclerosis; *κ*, 0.64 [0.46–0.81] (radiologist 2)) and excellent (facet joint degeneration; ICC 0.96 [0.90–1.00] (radiologist 1); Table 3).

**Table 3:** Agreement of imaging findings between MR-derived and conventional CT

Parameter	T1SGRE and conventional CT		UTE and conventional CT	
	Radiologist 1	Radiologist 2	Radiologist 1	Radiologist 2
Fractures				
Genant classification (*κ*)	0.92 [0.83–1.00]	0.92 [0.83–1.00]	0.89 [0.79–0.99]	0.69 [0.52–0.85]
Anterior height vertebral body (ICC)	0.99 [0.99–1.00]	0.99 [0.99–1.00]	0.90 [0.81–0.95]	0.88 [0.78–0.94]
Posterior height vertebral body (ICC)	0.99 [0.99–1.00]	0.99 [0.99–1.00]	0.89 [0.80–0.94]	0.90 [0.80–0.94]
AO/Magerl fracture classification (*κ*)	0.90 [0.76–1.00]	0.89 [0.75–1.00]	0.78 [0.58–0.98]	0.70 [0.49–0.92]
Fracture age (*κ*)	0.87 [0.78–0.96]	0.81 [0.71–0.92]	0.66 [0.53–0.80]	0.64 [0.49–0.79]
Degenerative changes				
Anterior disc height (ICC)	0.99 [0.98–0.92]	0.98 [0.97–0.99]	0.95 [0.92–0.97]	0.97 [0.94–0.98]
Posterior disc height (ICC)	0.97 [0.95–0.98]	0.99 [0.97–0.99]	0.96 [0.93–0.97]	0.96 [0.93–0.98]
Neuroforamen AP diameter (ICC)	0.95 [0.89–0-97]	0.81 [0.62–0.90]	0.91 [0.85–0.95]	0.79 [0.61–0.88]
Spondylolisthesis (ICC)	0.99 [0.99–1.00]	0.99 [0.99–1.00]	0.99 [0.99–1.00]	0.98 [0.97–0.99]
Osteophytes (κ)	0.91 [0.82–1.00]	0.87 [0.78–0.97]	0.78 [066–0.90]	0.73 [0.59–0.87]
Sclerosis (κ)	0.68 [0.48–0.88]	0.64 [0.46–0.81]	0.52 [0.32–0.72]	0.43 [0.26–0.60]
Facet joint degeneration (κ)	0.96 [0.90–1.00]	0.88 [0.78–0.97]	0.66 [0.48–0.84]	0.67 [0.53–0.81]

Data are given with 95% confidence intervals. *κ*, weighted Cohen’s kappa; *ICC*, intraclass correlation coefficient

Between UTE images and CT, agreement for quantitative parameters was generally lower, with ICCs ranging from 0.79 (0.61–0.88) (neuroforaminal AP diameter; radiologist 2) to 0.99 (0.99–1.00) (spondylolisthesis; radiologist 1; Table 3, Fig. 4). Analogously, agreement between UTE images and CT for ordinal-scale parameters was generally lower, with *κ* ranging between 0.43 (0.26–0.60) (sclerosis; radiologist 2) and 0.89 (0.79–0.99) (Genant classification; radiologist 1).

Inter-reader agreement ranged between substantial (sclerosis as evaluated on UTE images; κ, 0.52 [0.60–0.90]) and excellent (e.g., posterior height of the vertebral body evaluated on T1SGRE images and CT; ICCs, 0.99 [0.99–1.00], respectively; Table 4).

**Table 4:** Inter-reader agreement of imaging findings between radiologist 1 and 2

Parameter	T1SGRE	Conventional CT	UTE
Fractures			
Genant classification (*κ*)	0.95 [0.87–1.00]	0.95 [0.87–1.00]	0.77 [0.63–0.92]
Anterior height vertebral body (ICC)	0.98 [0.96–0.99]	0.98 [0.96–0.99]	0.97 [0.95–0.99]
Posterior height vertebral body (ICC)	0.99 [0.99–1.00]	0.99 [0.99–1.00]	0.99 [0.98–0.99]
AO/Magerl fracture classification (*κ*)	0.81 [0.63–0.99]	0.95 [0.84–1.00]	0.77 [0.56–0.98]
Fracture age (*κ*)	0.85 [0.76–0.95]	0.95 [0.85–1.00]	0.81 [0.69–0.93]
Degenerative changes			
Anterior disc height (ICC)	0.99 [0.98–0.99]	0.99 [0.97–0.99]	0.98 [0.96–0.99]
Posterior disc height (ICC)	0.98 [0.97–0.99]	0.98 [0.96–0.99]	0.97 [0.95–0.98]
Neuroforamen AP diameter (ICC)	0.93 [0.88–0.96]	0.87 [0.76–0.93]	0.98 [0.97–0.99]
Spondylolisthesis (ICC)	1.00 [1.00–1.00]	1.00 [1.00–1.00]	0.99 [098–0.99]
Osteophytes (*κ*)	0.82 [0.71–0.93]	0.83 [0.71–0.95]	0.81 [0.69–0.93]
Sclerosis (*κ*)	0.75 [0.60–0.90]	0.79 [0.67–0.91]	0.52 [0.29–0.75]
Facet joint degeneration (*κ*)	0.83 [0.73–0.94]	0.91 [0.84–1.00]	0.69 [0.53–0.86]

Data are given with 95% confidence intervals. *κ*, weighted Cohen’s kappa; *ICC*, intraclass correlation coefficient

## Discussion

In this analysis, we found a substantial to perfect diagnostic performance of T1SGRE-derived CT-like images and UTE with conventional CT for the identification of vertebral fractures. For the morphological assessment of fractures and degenerative bone changes, a substantial to perfect agreement was found as well as a robust image quality. Diagnostic performance of UTE, agreement between UTE and CT, and diagnostic image quality of UTE were generally lower. Inter-observer agreement was substantial-to-perfect for all modalities. Currently, patients with pathologies of the spine are regularly examined with CT and MR imaging to evaluate osseous and soft-tissue components of degenerative changes and fractures. For patients as well as from an economic perspective, it would be desirable to acquire all information in one examination. The CT-like images based on the T1SGRE sequence could enable MRI to reliably assess bone changes.

In particular, distance measurements were almost identical to measurements on conventional CT. Also, critical categorical variables such as the Genant and the AO/Magerl classifications showed an excellent agreement. By contrast, the extent of diffuse sclerosis still agreed substantially between T1SGRE-derived and conventional CT images, but this finding was affected by the tissue contrast on the T1SGRE sequence: Edema-like signal changes as occurring e.g. in Modic I changes induce a T1w signal decrease, and on intensity-inverted images, this might be indiscernible from sclerotic bone changes. Images should therefore always be read in combination with a fluid-sensitive pulse sequence.

Similarly, T1SGRE is not able to differentiate between bone and ligaments which both appear bright on grayscale-inverted reformatted images, as seen in Fig. 2. This must not be misinterpreted as ligament calcifications, and in the same context, the sensitivity of the proposed method for the assessment of ligament ossifications occurring e.g. in diffuse idiopathic skeletal hyperostosis must be expected to be low. Finally, gas accumulations in the intervertebral disc (“ vacuum phenomenon”) are devoid of signal and thus, bright on inverted reformatted images, not to be misinterpreted as calcifications.

The agreement between UTE and CT was lower for all assessed parameters, as well as the diagnostic image quality. Previously, UTE imaging was used to assess cortical bone in specimens [19], and trabecular bone in volunteers [18]. While showing convincing SNR in volunteers, the acquisition duration was > 9 min. In another study assessing simulated spondylolysis in cadaveric spine specimens, diagnostic confidence of UTE imaging was comparable to CT [32]. By contrast, the study presented here is based on clinical subjects. SNR and signal homogeneity in the thoracolumbar spine were not optimal, and UTE was prone to pulsation and movement artefacts. Moreover, UTE as a non-Cartesian imaging method is sensitive to off-resonance blurring induced by B_0_ inhomogeneities and fat chemical shift. Most importantly, the presently employed UTE imaging protocol needed a large field-of-view and substantial oversampling to avoid folding and the slice thickness was twice the thickness of T1SGRE to maintain acceptable acquisition durations. To maintain a primarily PD-weighted contrast, a small flip angle had to be used which reduced SNR. Also, to achieve an acceptable acquisition duration and advanced methods of improving short T2 contrast such as inversion recovery or subtraction methods were not used here. The use of long-T2 suppression techniques has to be evaluated in the future. Of note, UTE imaging has potential advantages regarding tissue contrast: It may be highly useful e.g. for assessing the cartilage endplate [33] and to differentiate ligaments and calcified structures. ZTE pulse sequences, on the other hand, generate real PD-weighted tissue contrast and have been successfully applied to the shoulder, hip, skull, and cervical spine [20, 34–36]. How they perform in the thoracolumbar spine in comparison to T1SGRE should be evaluated in future studies.

Particularly in the context of emergency care, MRI compared to CT is usually less accessible, associated with higher costs, longer examination duration and requires a good patient compliance. Therefore, the application of the proposed method may be limited to centers with necessary resources and/ or to patients which will undergo an MRI examination in any case.

However, it has to be noted that conventional CT, which served as the standard of reference for the assessment of morphologic bone changes here, may miss “occult vertebral fractures”, i.e., traumatic injuries without significant morphologic changes but with the presence of a “bone bruise”, i.e. edema-equivalent signal changes in the bone marrow that can be detected on fluid-sensitive MRI sequences [37, 38]. In this study, no patient with this trauma pattern was included, but it may be assumed that the combination of clinically established MRI sequences including STIR and the proposed sequences for dedicated bone assessment may be the most sensitive sensible option for the detection of vertebral injuries.

This study has limitations. First, the acquisition of the proposed gradient duty-cycle intensive pulse sequences requires a 3-T MR scanner with state-of-the-art gradient coils. On older scanners, examination duration might be prolonged due to increased minimum TR. Furthermore, particularly T1SGRE and less so UTE are sensitive to metal artifacts and thus, in patients with metallic implants, CT or CT myelography may still be the preferred diagnostic method.

Moreover, no patients with more severe fracture patterns, i.e., distraction and translation injuries, were included in this study, since these patients regularly undergo surgery immediately after CT. Since all morphometric parameters assessed here showed a substantial to perfect agreement, it could be assumed that this would apply to more severe injury patterns just as well; however, this needs to be evaluated in future studies. Analogously, in this first assessment, we did not include patients with pathologic fractures or bone metastases. Whether or not the used sequences are equivalent to CT or may even add diagnostic value in these cases (due to perfect alignment with other MR sequences in the protocol) has yet to be evaluated.

In summary, CT-like images obtained from a T1SGRE sequence showed a substantial to perfect agreement with conventional CT for the assessment of vertebral fractures and degenerative bony changes. Agreement between UTE imaging and CT was substantial but generally lower due to primarily less robust UTE image quality. Therefore, in specific cases, adding the T1SGRE sequence to spine MR examination protocols could render additional CT examinations obsolete in the future, spare additional examinations, and reduce radiation doses and costs.

## Abbreviations

T1SGRE: T1-weighted spoiled gradient-echo
UTE: Ultra-short echo time
ZTE: Zero echo time
